# Supplementary material for: Interferon-β Activity Is Affected by S100B Protein
Source: Int J Mol Sci. 2022 Feb 11;23(4):1997. doi: 10.3390/ijms23041997 (PMC8877046; doi:10.3390/ijms23041997)
Supplement: Supplementary file 1 [file ijms-23-01997-s001.zip › ijms-1505477-supplementary.pdf]

Interferon Beta Activity Is Affected by S100B Protein

Alexey S. Kazakov [1], Alexander D. Sofin [1], Nadezhda V. Avkhacheva [1], Evgenia I. Deryusheva [1], Victoria A. Rastrygina [1], Maria E. Permyakova [1], Vladimir N. Uversky [2,*], Eugene A. Permyakov [1] and Sergei E. Permyakov [1,*]

[1] Institute for Biological Instrumentation, Pushchino Scientific Center for Biological Research of the Russian Academy of Sciences, Institutskaya str., 7, Pushchino, Moscow Region 142290, Russia; fenixfly@yandex.ru (A.S.K.); AlSofin@mail.ru (A.D.S.); avkhacheva@gmail.com (N.V.A.); janed1986@ya.ru (); certusfides@gmail.com (V.A.R.); mperm1977@gmail.com (M.E.P.); epermyak@yandex.ru (E.A.P.); permyakov.s@gmail.com (S.E.P.)

[2] Department of Molecular Medicine and USF Health Byrd Alzheimer's Research Institute, Morsani College of Medicine, University of South Florida, Tampa, FL 33612, USA

* Correspondence: vuversky@usf.edu (V.N.U.); permyakov.s@gmail.com (S.E.P.); Tel.: +7-(495)-143-7741 (S.E.P.); Fax: +7-(4967)-33-05-22 (S.E.P.)

Table S1. List of the human diseases associated with IFN-β and S100B protein, according to DisGeNET database (http://www.disgenet.org). PubMed identifiers of the references confirming the protein-disease associations are indicated.

№	Disease	IFN-β	S100B
1	Glioblastoma	29557060; 27637889; 28098415; 29159280; 28912136; 27194146; 25920530; 26441059; 26397698; 24526161; 23384727; 21805051; 20606645; 19197327; 15455376; 14965379; 1501894; 1998958; 1653364	21714070; 18577219
2	Cutaneous Melanoma	29136453	21654685; 8225871
3	Malignant neoplasm of urinary bladder	11948141; 2297754	17970044
4	Secondary malignant neoplasm of liver	28008721; 21958207; 14977858; 12405292; 11435460	29387968; 22011044
5	Atrial Fibrillation	26056004	31118294; 31575426
6	Lupus Erythematosus, Discoid	29466691	31271591
7	Dermatomyositis	30101523; 27564228; 23018857; 21177291; 21670437; 17968926	30157932
8	Ischemic stroke	31810024; 31100209; 30320448; 12973019	29384222; 29343763; 28598212
9	Colon Carcinoma	31348591; 29496994; 18035482; 1586975	31266264
10	Primary malignant neoplasm	30864696; 30613977; 31167402; 31348591; 30679806; 31036052; 30446226; 29750542; 29158470; 27637889; 29229854; 28214639; 28912136; 28129467; 26597133; 27077807; 26880763; 27496321; 27532609; 24141111; 23403306; 22522623; 22842821; 21226949; 22106296	30710273; 31266264; 29345293
11	Seizures	12432978	30173303; 17955279
12	Schizophrenia	26133963	31786729; 30144068; 30618856; 28435992; 29164477; 29875007; 27863321; 22019077; 22146151; 22146151; 21112154; 21714070; 21070816; 21070816; 20398908; 20398908; 19103440; 20093730; 17982903; 17525977; 17043297; 17062375;

			15670788; 15670788
13	Non-Small Cell Lung Carcinoma	30622322; 26431376; 23658645	30455129; 22286962; 19118029
14	Glioma	31638255; 31100209; 26252165; 25175315; 24671169; 24526161; 22938469; 21805051; 19893595; 18220319; 16343929; 14965379; 15546502; 11856487; 10982152; 10395172; 9875676; 1998958	30076898; 8750867
15	Coronary heart disease	25882064	29183208; 30966831; 28672919
16	Memory impairment	31152860	31202867; 30590973; 11222062
17	Breast Carcinoma	31167402; 27207649; 27077807; 26716512; 25884794; 24141111; 18608205; 18608205; 9059994	29345293; 20145129
18	Brain Neoplasms	23384727; 21805051; 19197327; 1501894	30455129
19	Cerebrovascular accident	30320448; 12973019	31387626; 30144068; 31356182; 30229393; 29792379; 29249591; 16960091
20	Cone-Rod Dystrophy 2	17125797	29959890
21	Carcinoma of lung	30622322; 31428903; 30683475; 30446226; 26351076; 25486572; 19893592; 19564073; 2560625	21861268
22	Septicemia	30770245; 31500303; 29741098; 30319651; 28533774	27436624
23	Depressed mood	28497489; 28245725; 29055445; 28500224	30745657; 31100426; 30506371; 31493698; 30278520; 28594820; 19497163; 16797859; 8804639; 8840925
24	Sepsis	30770245; 31500303; 29741098; 30319651; 28533774	27436624
25	nervous system disorder	27613121	31067852; 29329729
26	Neoplasm Metastasis	31070806; 30213181; 29202279; 28129467; 26716512; 26102027; 20197756; 12405292; 11948141; 11498771; 10029078; 9815626	31278360; 29345293; 17264538
27	Malignant neoplasm of lung	30683475; 30622322; 30446226; 26351076; 25486572; 19893592; 19564073; 2560625	21861268
28	Malignant neoplasm of breast	31167402; 26716512; 27077807; 25884794; 24141111; 18608205;	29345293; 20145129

		18608205	
29	Polymyositis	21670437; 17968926	30157932
30	Neuroblastoma	27637889; 26887385; 28882441; 26943965; 25920530; 25086905; 18726131; 16115947	21714070; 19216736; 9458088; 8733171
31	Brain Diseases	12618863; 8615598	31009650; 21112154; 18454941; 18454941; 17705023
32	estrogen receptor-negative breast cancer	8669823	29345293
33	Status Epilepticus	28166388	29506554; 29680360; 28444637
34	Lupus Erythematosus	29466691	31271591
35	melanoma	31356866; 30864696; 30213181; 31070806; 28649738; 27677689; 28624449; 28877249; 29136453; 27399807; 26102027; 26054674; 25728676; 23370279; 23358428; 23018621; 22555508; 21723255; 21846298; 21493591; 18945721; 16505900; 16928243; 16343929; 12096926	29135116; 30710305; 29849947; 29345293; 28212812; 27633490; 26659191; 26288839; 24627490; 24490776; 23375094
36	Neuromyelitis Optica	31136907; 28600575; 23434493; 17125797	30426010; 19372295
37	Tumor Cell Invasion	29618645; 28317173; 25486572	31266264; 28849099; 22286962; 21861268
38	Depressive disorder	28497489; 28500224; 28245725; 29055445	30745657; 31100426; 30506371; 31493698; 30278520; 28594820; 19497163; 19497163; 16797859; 16797859; 8804639; 8840925; 8804639; 8840925
39	Stomach Carcinoma	22159640	25266115
40	Mental Depression	28497489; 29055445; 28500224; 28245725	30745657; 30506371; 31493698; 31100426; 30278520; 28594820; 16797859; 16797859; 8804639; 8840925; 8804639; 8840925
41	Impaired cognition	31233762; 31152860; 29063244; 21208197	31683478; 31402961; 29957487; 29523900; 27641367; 27863321; 17579612; 11222062
42	Mental deterioration	21208197	17579612
43	Forgetful	31152860	30590973; 11222062

44	Adult type dermatomyositis	30101523; 27564228; 23018857; 21177291; 21670437	30157932
45	Psoriasis	27438769	29186242; 17719207
46	Lupus Vulgaris	29466691	31271591
47	Human immunodeficiency virus (HIV) II infection category B1	28009639; 10954903; 10446920	28852071
48	Secondary Neoplasm	20197756	31278360
49	Degenerative polyarthritis	23369825	31100426; 30280200
50	leukemia	2295067	8677743
51	Vitiligo	28752785	28647026; 28212812
52	Leukemia, T-Cell	28768861	8381310; 2896028
53	Childhood Astrocytoma	19267105; 8103050; 1906070	19147496
54	Astrocytoma	19267105; 15455376; 8174086; 8103050; 1906070	19147496; 2186177
55	Lupus Erythematosus, Systemic	31153744; 29466691; 30201809; 28471483; 24561305; 24644022; 22859983; 17968925	31271591; 30392117; 30376438; 30282561; 17955279
56	Primary malignant neoplasm of lung	30683475; 30622322; 30446226; 26351076; 25486572; 19893592; 19564073; 2560625	21861268
57	Malignant Neoplasms	30864696; 30679806; 31348591; 31036052; 31167402; 30613977; 30446226; 29158470; 29750542; 28214639; 26597133; 29229854; 28912136; 28129467; 27637889; 27532609; 26880763; 27496321; 27077807; 24141111; 23403306; 22522623; 22842821; 22106296; 21226949	30732223; 30710281; 31266264; 29345293; 30366122; 29987748; 28051137; 25614008; 19351828; 18705642
58	Adult Glioblastoma	29557060; 28098415; 29159280; 27637889; 27194146; 26441059; 25920530; 24526161; 19197327; 1998958	21714070
59	Virus Diseases	31495880; 30275539; 31461630; 31500303; 29979632; 31379819; 31043529; 31594636; 30522781; 31603951; 30930359; 31154625; 30995506; 29559569; 29046453; 30089112; 30333836; 30298349; 30569290; 29611236; 28589097; 29258190; 28573616; 27983470; 29138248	29953169

60	VITILIGO-ASSOCIATED MULTIPLE AUTOIMMUNE DISEASE SUSCEPTIBILITY 1 (finding)	28752785	28647026; 28212812
61	Diabetes Mellitus, Insulin-Dependent	16504056	31067852; 29484801
62	Inflammatory Bowel Diseases	27220814	30144068; 19558426
63	Obesity	29914624; 22951153	31363816; 28174179
64	Seizures, Focal	12432978	30173303
65	Coronary Artery Disease	25882064	30783474
66	Glioblastoma Multiforme	29557060; 29159280; 28912136; 28098415; 27637889; 27194146; 25920530; 26441059; 24526161; 23384727; 20606645; 19197327; 18714312; 14965379; 1653364; 1998958	21714070; 18577219
67	Multiple Sclerosis	30610426; 31467033; 31732227; 31606972; 31649263; 31793659; 31522451; 30401571; 30763907; 30684504; 29440323; 30289355; 31209101; 30592627; 30692524; 31589278; 31331574; 31202258; 31517556; 31235738; 30917079; 31565644; 30315270; 31354720; 30594597	30368653; 30319357; 19372295; 12355425
68	Childhood Neuroblastoma	26887385; 28882441; 27637889; 26943965; 25920530; 25086905; 18726131	21714070; 19216736; 9458088; 8733171
69	Amyloidosis	30222725	29259249; 24586351; 19705461; 16709678
70	Encephalitis, St. Louis	31153744	30376438
71	Meningioma	27873050	17020600; 15492810
72	Alzheimer's Disease	30610591; 31152860; 31233762; 30076830; 30222725; 24262201	31683478; 30649628; 30144068; 31217937; 31281238; 29963623; 29681765; 30045751; 28582866; 29386995; 27585561; 20953641; 21080947; 19705461; 19205880; 16797859; 15290893; 15126113; 12505619; 11754997; 11222062; 10617132; 10381557

73	Bacterial Infections	29190678; 28422568; 28461567	28815036
74	Adult T-Cell Lymphoma/Leukemia	28768861	8381310
75	Central neuroblastoma	27637889; 26887385; 28882441; 26943965; 25920530; 25086905; 18726131	21714070; 19216736; 9458088; 8733171
76	Metastatic melanoma	21235385; 10942517	31704599; 29792379; 25586991; 16179868
77	Pain	29394383	31824318
78	Infection	19201909; 16984921; 16002711; 15280450; 15163721	17624933
79	Secondary malignant neoplasm of lymph node	30672717; 23358428	23519055
80	Malignant Pleural Mesothelioma	19715403; 17671130	28377727
81	Purpura, Thrombotic Thrombocytopenic	28791286	29572925
82	Malignant neoplasm of stomach	22159640	25266115
83	Childhood Glioblastoma	29557060; 28098415; 29159280; 27637889; 27194146; 26441059; 25920530; 24526161; 19197327; 1998958	21714070
84	Neoplasms	31512776; 31776268; 30683475; 31070806; 31627888; 30848981; 31036052; 30393160; 31358054; 31431457; 30675668; 30679806; 30622322; 29750542; 30012853; 29849115; 28912136; 28337378; 28514874; 27188205; 28578991; 28098415; 28483787; 27399807; 26880763	31704599; 29135116; 30076898; 29519923
85	Hepatitis	30594597; 22106296	18173564
86	Malignant neoplasm of ovary	11896621	31033462; 30558666; 29345293
87	Tumor Progression	31821828; 28849002	20587415
88	Optic Atrophy	21987543	29959432

Table S2. List of the human diseases associated with IFN-β and S100B protein, according to Open Targets Platform database (https://www.opentargets.org), and corresponding association scores (https://docs.targetvalidation.org/getting-started/scoring). Diseases with association scores for the both proteins exceeding 0.1 are highlighted in yellow.

№	Disease	IFN-β	S100B
1	cutaneous melanoma	0.038	0.367
2	brain neoplasm	0.788	0.168
3	Ischemic stroke	0.015	0.069
4	ischemia	0.053	0.089
5	infectious meningitis	0.021	0.059
6	dilated cardiomyopathy	0.256	0.059
7	HIV-1 infection	0.042	0.103
8	brain injury	0.046	0.081
9	X-linked adrenoleukodystrophy	0.046	0.033
10	non-small cell lung carcinoma	0.646	0.076
11	colorectal carcinoma	0.063	0.1
12	idiopathic pulmonary fibrosis	0.031	0.013
13	hyperplasia	0.05	0.027
14	HIV infection	0.058	0.107
15	esophageal squamous cell carcinoma	0.04	0.017
16	colon carcinoma	0.06	0.095
17	Chronic mucocutaneous candidosis	0.021	0.015
18	amyotrophic lateral sclerosis	0.036	0.16
19	ulcer disease	0.063	0.036
20	unipolar depression	0.083	0.099
21	viral disease	0.225	0.122
22	Sleep Disorder	0.025	0.058
23	intoxication	0.01	0.074
24	Porphyria cutanea tarda	0.04	0.138
25	Parkinson's disease	0.041	0.067
26	kidney failure	0.016	0.044
27	paraneoplastic neurologic syndrome	0.01	0.008

28	Weight loss	0.022	0.02
29	colorectal adenocarcinoma	0.044	0.067
30	complication	0.078	0.069
31	psychosis	0.013	0.17
32	pneumonia	0.049	0.017
33	influenza	0.191	0.015
34	Charcot-Marie-Tooth disease type 1A	0.198	0.022
35	wet macular degeneration	0.046	0.01
36	adverse effect	0.077	0.013
37	arterial occlusion	0.025	0.044
38	fibrosis	0.055	0.018
39	glioma	0.788	0.168
40	neurofibroma	0.006	0.054
41	Coma	0.012	0.028
42	optic neuritis	0.044	0.012
43	stroke	0.13	0.121
44	hypertension	0.076	0.144
45	invasive breast ductal carcinoma	0.009	0.043
46	peripheral neuropathy	0.346	0.19
47	asphyxia	0.004	0.04
48	multiple sclerosis	0.119	0.064
49	small cell lung carcinoma	0.033	0.051
50	meningoencephalitis	0.012	0.01
51	epilepsy	0.308	0.081
52	myositis	0.076	0.032
53	melanoma	0.188	0.382
54	paraplegia	0.255	0.016
55	atherosclerosis	0.052	0.018
56	type II hypersensitivity reaction disease	0.258	0.101
57	exhaustion	0.016	0.031
58	psoriasis vulgaris	0.011	0.015

59	pulmonary arterial hypertension	0.075	0.031
60	neuropathic pain	0.013	0.014
61	Neurodegeneration	0.048	0.055
62	Headache	0.073	0.041
63	Proteinuria	0.065	0.037
64	memory impairment	0.022	0.03
65	amyloidosis	0.013	0.074
66	Sepsis	0.081	0.057
67	Fever	0.065	0.031
68	animal viral hepatitis	0.019	0.026
69	Brain atrophy	0.036	0.028
70	ischemia reperfusion injury	0.023	0.032
71	glioblastoma multiforme	0.676	0.032
72	head and neck squamous cell carcinoma	0.054	0.5
73	primary progressive multiple sclerosis	0.056	0.045
74	skin neoplasm	0.188	0.615
75	dizziness	0.012	0.004
76	squamous cell carcinoma	0.056	0.505
77	mucous membrane pemphigoid	0.04	0.01
78	neurodegenerative disease	0.354	0.172
79	immunophenotype	0.03	0.019
80	asthma	0.165	0.004
81	lung disease	0.649	0.1
82	insomnia	0.011	0.033
83	anaplastic astrocytoma	0.045	0.011
84	AIDS dementia	0.017	0.011
85	nervous system disease	0.85	0.218
86	inflammatory bowel disease	0.059	0.063
87	metastatic melanoma	0.05	0.06
88	Small for gestational age	0.01	0.028
89	hepatocellular carcinoma	0.112	0.01

90	heart disease	0.283	0.077
91	Ascites	0.051	0.011
92	mental or behavioural disorder	0.331	0.177
93	dysplasia	0.029	0.011
94	colon adenocarcinoma	0.016	0.029
95	nervousness	0.066	0.064
96	Myelitis	0.017	0.011
97	myocardial infarction	0.024	0.049
98	tropical spastic paraparesis	0.044	0.024
99	anxiety disorder	0.029	0.061
100	chronic hepatitis C virus infection	0.079	0.013
101	allergy	0.027	0.017
102	lung cancer	0.647	0.082
103	necrosis	0.064	0.019
104	severe cutaneous adverse reaction	0.05	0.018
105	systemic lupus erythematosus	0.099	0.087
106	kidney injury	0.01	0.011
107	aspergillosis	0.013	0.035
108	obesity	0.198	0.063
109	stomach neoplasm	0.064	0.01
110	hypoxia	0.046	0.084
111	ovarian carcinoma	0.697	0.059
112	Alzheimer's disease	0.044	0.067
113	urethral intrinsic sphincter deficiency	0.043	0.023
114	acute myeloid leukemia	0.035	0.063
115	Encephalopathy	0.024	0.04
116	cognitive disorder	0.284	0.176
117	psoriatic arthritis	0.036	0.017
118	nervous system injury	0.059	0.081
119	fetal growth restriction	0.012	0.052
120	Chronic granulomatous disease	0.011	0.006

121	head injury	0.015	0.083
122	encephalitis	0.058	0.046
123	mood disorder	0.083	0.102
124	Myasthenia gravis	0.035	0.011
125	metastatic neoplasm	0.05	0.06
126	Retinoblastoma	0.069	0.016
127	infertility	0.078	0.062
128	bacterial disease	0.156	0.045
129	neuropathy	0.346	0.19
130	urinary bladder cancer	0.048	0.01
131	asphyxia neonatorum	0.038	0.073
132	inflammation	0.102	0.123
133	cirrhosis of liver	0.053	0.016
134	vascular disease	0.234	0.158
135	migraine without aura	0.018	0.039
136	type I diabetes mellitus	0.048	0.039
137	peritonitis	0.038	0.011
138	renal cell adenocarcinoma	0.078	0.017
139	cerebellar ataxia	0.253	0.063
140	heart failure	0.015	0.047
141	congenital heart disease	0.013	0.049
142	experimental autoimmune encephalomyelitis	0.113	0.015
143	attention deficit hyperactivity disorder	0.01	0.058
144	type IV hypersensitivity disease	0.235	0.025
145	liver disease	0.115	0.159
146	chronic hepatitis	0.046	0.012
147	adenocarcinoma	0.778	0.048
148	edema	0.015	0.026
149	schizophrenia	0.01	0.168
150	Hyperglycemia	0.012	0.017

151	infectious disease	0.335	0.124
152	migraine disorder	0.052	0.062
153	Myelopathy	0.015	0.024
154	relapsing-remitting multiple sclerosis	0.11	0.031
155	breast cancer	0.725	0.029
156	Hypothermia	0.046	0.079
157	hemorrhage	0.075	0.076
158	viral encephalitis	0.056	0.044
159	Abnormality of mitochondrial metabolism	0.011	0.018
160	cerebral artery occlusion	0.024	0.044
161	cancer	0.838	0.619
162	celiac disease	0.011	0.053
163	neoplasm	0.85	0.633
164	Spinal cord injury	0.052	0.043
165	diabetes mellitus	0.05	0.09
166	osteosarcoma	0.044	0.01
167	vasculitis	0.043	0.014
168	Fanconi anemia complementation group E	0.014	0.026
169	Herpetic encephalitis	0.015	0.017
170	neuromyelitis optica	0.051	0.028
171	medulloblastoma	0.042	0.071
172	Gliosis	0.017	0.078
173	neuroblastoma	0.082	0.047
174	septic shock	0.05	0.03
175	pulmonary edema	0.011	0.016
176	psychotic symptoms	0.01	0.039
177	colitis	0.058	0.054
178	transient ischemic attack	0.013	0.021
179	Sarcoidosis	0.079	0.02
180	hepatitis C virus infection	0.079	0.029
181	brain edema	0.019	0.06

182	chronic kidney disease	0.004	0.037
183	Uveal Melanoma	0.037	0.033
184	Ataxia-telangiectasia	0.031	0.05
185	Cognitive impairment	0.025	0.062
186	Vitiligo	0.017	0.077
187	osteoarthritis	0.04	0.043
188	injury	0.116	0.144
189	central nervous system disease	0.85	0.214
190	Nausea	0.01	0.013
191	Frontotemporal dementia	0.011	0.011
192	Meningioma	0.041	0.044
193	respiratory failure	0.067	0.018
194	Tuberous sclerosis	0.029	0.011
195	encephalomyelitis	0.228	0.064
196	Growth delay	0.021	0.026
197	Potassium-aggravated myotonia	0.044	0.018
198	brain infarction	0.041	0.067
199	Shock	0.044	0.05
200	Peripheral demyelination	0.058	0.027
201	grade III glioma	0.03	0.01
202	substance dependence	0.062	0.06
203	Guillain-Barre syndrome	0.052	0.041
204	psoriasis	0.056	0.048
205	cerebral infarction	0.04	0.067
206	nicotine dependence	0.06	0.018
207	Mental deterioration	0.015	0.034
208	Seizure	0.021	0.082
209	ulcerative colitis	0.05	0.055
210	osteonecrosis	0.048	0.054
211	diarrheal disease	0.035	0.014
212	toxic encephalopathy	0.024	0.063

213	delirium	0.01	0.147
214	breast carcinoma	0.709	0.062
215	pain	0.06	0.064
216	keratitis	0.048	0.014
217	chronic obstructive pulmonary disease	0.066	0.08
218	astrocytoma	0.676	0.036
219	cardiac hypertrophy	0.011	0.019
220	Paroxysmal exertion-induced dyskinesia	0.012	0.008
221	dementia (disease)	0.284	0.074
222	Duchenne muscular dystrophy	0.033	0.013
223	non-small cell lung adenocarcinoma	0.02	0.038
224	polyneuropathy	0.049	0.013
225	subarachnoid hemorrhage	0.034	0.066

Figure S1. Multiple sequence alignment of human S100B, S100A1, S100A4, S100A6, and S100P proteins by Clustal Omega.

```
sp|P25815|S100P_HUMAN      -MTELETAMGMIIDVFSRYSGSEGSTQTLTKGELKVLMEKELPGFLQSGKDKDAVDKLLK     59
sp|P23297|S10A1_HUMAN      MGSELETAMETLINVFHAHSGKEGDKYKLSKKELKELLQTELSGFLDAQKDVDAVDKVMK     60
sp|P26447|S10A4_HUMAN      MACPLEKALDVMVSTFHKYSGKEGDKFKLNKSELKELLTRELPSFLGKRTDEAAFQKLMS     60
sp|P06703|S10A6_HUMAN      MACPLDQAIGLLVAIFHKYSGREGDKHTLSKKELKELIQKELT--IGSKLQDAEIARLME     58
sp|P04271|S100B_HUMAN      -MSELEKAMVALIDVFHQYSGREGDKHKLKKSELKELINNELSHFLEEIKEQEVVDKVME     59
                               *: *:  ::  *  :** **.. .*.* *** *:  **   :    :   . :::.

sp|P25815|S100P_HUMAN      DLDANGDAQVDFSEFIVFVAAITSACHKYFEKAGLK-----      95
sp|P23297|S10A1_HUMAN      ELDENGDGEVDFQEYVVLVAALTVACNNFFWENS-------      94
sp|P26447|S10A4_HUMAN      NLDSNRDNEVDFQEYCVFLSCIAMMCNEFFEGFPDKQPRKK      101
sp|P06703|S10A6_HUMAN      DLDRNKDQEVNFQEYVTFLGALALIYNEALKG---------      90
sp|P04271|S100B_HUMAN      TLDNDGDGECDFQEFMAFVAMVTTACHEFFEHE--------      92
                            ** : * : :*.*: .::. ::   :: :
```

```
Percent Identity Matrix - created by Clustal2.1

     1: sp|P25815|S100P_HUMAN  100.00   52.69   41.05   34.83   50.00
     2: sp|P23297|S10A1_HUMAN   52.69  100.00   48.94   43.33   57.61
     3: sp|P26447|S10A4_HUMAN   41.05   48.94  100.00   51.11   45.65
     4: sp|P06703|S10A6_HUMAN   34.83   43.33   51.11  100.00   39.33
     5: sp|P04271|S100B_HUMAN   50.00   57.61   45.65   39.33  100.00
```
